# Overcoming Multidrug Resistance via Photodestruction of ABCG2-Rich Extracellular Vesicles Sequestering Photosensitive Chemotherapeutics

**DOI:** 10.1371/journal.pone.0035487

**Published:** 2012-04-18

**Authors:** Vicky Goler-Baron, Yehuda G. Assaraf

**Affiliations:** The Fred Wyszkowski Cancer Research Laboratory, Department of Biology, Technion-Israel Institute of Technology, Haifa, Israel; Enzo Life Sciences, Inc., United States of America

## Abstract

Multidrug resistance (MDR) remains a dominant impediment to curative cancer chemotherapy. Efflux transporters of the ATP-binding cassette (ABC) superfamily including ABCG2, ABCB1 and ABCC1 mediate MDR to multiple structurally and functionally distinct antitumor agents. Recently we identified a novel mechanism of MDR in which ABCG2-rich extracellular vesicles (EVs) form in between attached neighbor breast cancer cells and highly concentrate various chemotherapeutics in an ABCG2-dependent manner, thereby sequestering them away from their intracellular targets. Hence, development of novel strategies to overcome MDR modalities is a major goal of cancer research. Towards this end, we here developed a novel approach to selectively target and kill MDR cancer cells. We show that illumination of EVs that accumulated photosensitive cytotoxic drugs including imidazoacridinones (IAs) and topotecan resulted in intravesicular formation of reactive oxygen species (ROS) and severe damage to the EVs membrane that is shared by EVs-forming cells, thereby leading to tumor cell lysis and the overcoming of MDR. Furthermore, consistent with the weak base nature of IAs, MDR cells that are devoid of EVs but contained an increased number of lysosomes, highly accumulated IAs in lysosomes and upon photosensitization were efficiently killed via ROS-dependent lysosomal rupture. Combining targeted lysis of IAs-loaded EVs and lysosomes elicited a synergistic cytotoxic effect resulting in MDR reversal. In contrast, topotecan, a bona fide transport substrate of ABCG2, accumulated exclusively in EVs of MDR cells but was neither detected in lysosomes of normal breast epithelial cells nor in non-MDR breast cancer cells. This exclusive accumulation in EVs enhanced the selectivity of the cytotoxic effect exerted by photodynamic therapy to MDR cells without harming normal cells. Moreover, lysosomal alkalinization with bafilomycin A1 abrogated lysosomal accumulation of IAs, consequently preventing lysosomal photodestruction of normal breast epithelial cells. Thus, MDR modalities including ABCG2-dependent drug sequestration within EVs can be rationally converted to a pharmacologically lethal Trojan horse to selectively eradicate MDR cancer cells.

## Introduction

A primary hindrance to successful cancer therapy remains the frequent emergence of drug resistance to structurally and functionally unrelated anticancer drugs, a phenomenon known as multidrug resistance (MDR) [1], [2], [3], [4], [5], [6]. Transporters of the ATP-Binding Cassette (ABC) superfamily including ABCB1 (P-gp), ABCC1 (MRP1) and ABCG2 (BCRP), function as ATP-dependent MDR efflux transporters. These efflux pumps form a unique defense network against multiple chemotherapeutic drugs as well as endogenous and exogenous cellular toxicants. Recently, we identified a novel modality of chemoresistance to multiple anticancer drugs mediated by ABCG2-rich extracellular vesicles (EVs) formed between neighbor carcinoma cells. Although we initially identified and characterized EVs in mitoxantrone-resistant breast cancer cells [7], [8], drug sequestration by EVs was also found in other tumor cell lines of distinct tissue origin including non-small lung cancer [9] and gastric carcinoma cells [8]. Overexpression of ABCG2 in the membrane of EVs resulted in cellular resistance to several cytotoxic drugs including mitoxantrone [10], topotecan and imidazoacridinones (IAs) [8], due to their dramatic sequestration and concentration in the lumen of EVs. Inhibition of ABCG2 transport activity with the specific transport inhibitors Ko143 or fumitremorgin C (FTC) abolished intravesicular accumulation, thereby resulting in restoration of drug sensitivity. We further found EVs to be apically localized, sealed structures, reinforced by an actin-based cytoskeleton and secluded from the extracellular milieu by the tight junction proteins occludin and ZO-1. Moreover, Ezrin-Radixin-Moesin (ERM) protein complex selectively localized to the border of the EVs membrane, suggesting a key role for ERM in MDR pump tethering to the actin cytoskeleton [8].

IAs are cytotoxic fluorochromes with hydrophobic weak base properties which have shown significant clinical activity against colorectal and breast cancers [11]. IAs bearing a hydroxyl group at one of the R1, R2, R3 positions in the proximal IA ring, including C-1371, C-1492 and C-1309 were recognized as ABCG2 transport substrates and were actively extruded from ABCG2-overexpressing MDR cells [12]. In contrast, IAs lacking a hydroxyl group such as C-1266 and C-1375 were not recognized by the MDR efflux pump ABCG2. IAs share a close structural similarity to mitoxantrone which is an established topoisomerase II inhibitor [13], hence suggesting similar cellular targets for cytotoxicity.

Topotecan, a water-soluble derivative of camptothecin, is a natural chromophoric anticancer drug that elicits its cytotoxic activity by stabilizing a covalent topoisomerase I-DNA complex, thereby inflicting a hindrance to the progression of the DNA replication fork with subsequent formation of lethal DNA adducts [14]. Topotecan is an established ABCG2 transport substrate [15], [16], [17] and is currently approved for the treatment of ovarian cancer and small-cell lung cancer. We have previously shown that topotecan highly accumulates in EVs in an ABCG2-dependent manner, thereby resulting in 25-fold resistance in MCF-7/MR cells relatively to their parental MCF-7 cells [8].

Photodynamic therapy (PDT), a treatment modality for cancer and non-malignant disorders, involves administration of a photosensitizer and an accurate delivery of light to the tumor mass or the target tissue. Once excited by light at an appropriate wavelength, the photosensitizer releases energy that is transferred to molecular oxygen thereby forming reactive oxygen species (ROS), which elicit a potent cytotoxic effect [18], [19]. Taking advantage of the inherent fluorescent properties of IAs we show here that IAs which are ABCG2 substrates highly concentrate within EVs in an ABCG2-dependent manner, similarly to topotecan. Moreover, photoexcitation of these photosensitizer-loaded EVs resulted in the rapid rupture of the membrane of EVs, a process that was mediated by ROS [20]. Moreover, since the membrane of EVs is an integral part of the plasma membrane, EVs-forming tumor cells were efficiently killed due to rapid cell lysis. In contrast, IAs which are not ABCG2 substrates and cannot be sequestered within EVs, highly accumulated in lysosomes due to their hydrophobic weak base nature. Upon illumination, rapid production of ROS resulted in lysosomal photodestruction, spill of multiple lysosomal hydrolytic enzymes into the cytoplasm, thereby leading to rapid cell death. In contradistinction to IAs, topotecan was neither detected in lysosomes of cancer cells (MCF-7) nor in lysosomes of normal breast epithelial cells (MCF-10A), but accumulated exclusively in ABCG2-rich EVs of MDR cancer cells (MCF-7/MR). This exclusive concentration within EVs allowed for the selective destruction of MDR cells without harming normal cells; this selective cytotoxic effect was due to the lack of topotecan accumulation in lysosomes and hence the lack of lysosomal photodestruction in normal cells. Collectively, our current findings establish that ABCG2-mediated sequestration and accumulation of certain photosensitizers within EVs markedly enhances the selectivity and the cytotoxic activity exerted by PDT, thereby rendering these EVs a pharmacological photosensitive lethal Trojan horse for the future overcoming of MDR cancers.

## Results

### IAs are highly concentrated in ABCG2-rich EVs formed in MCF-7/MR cells

**Figure 1:**
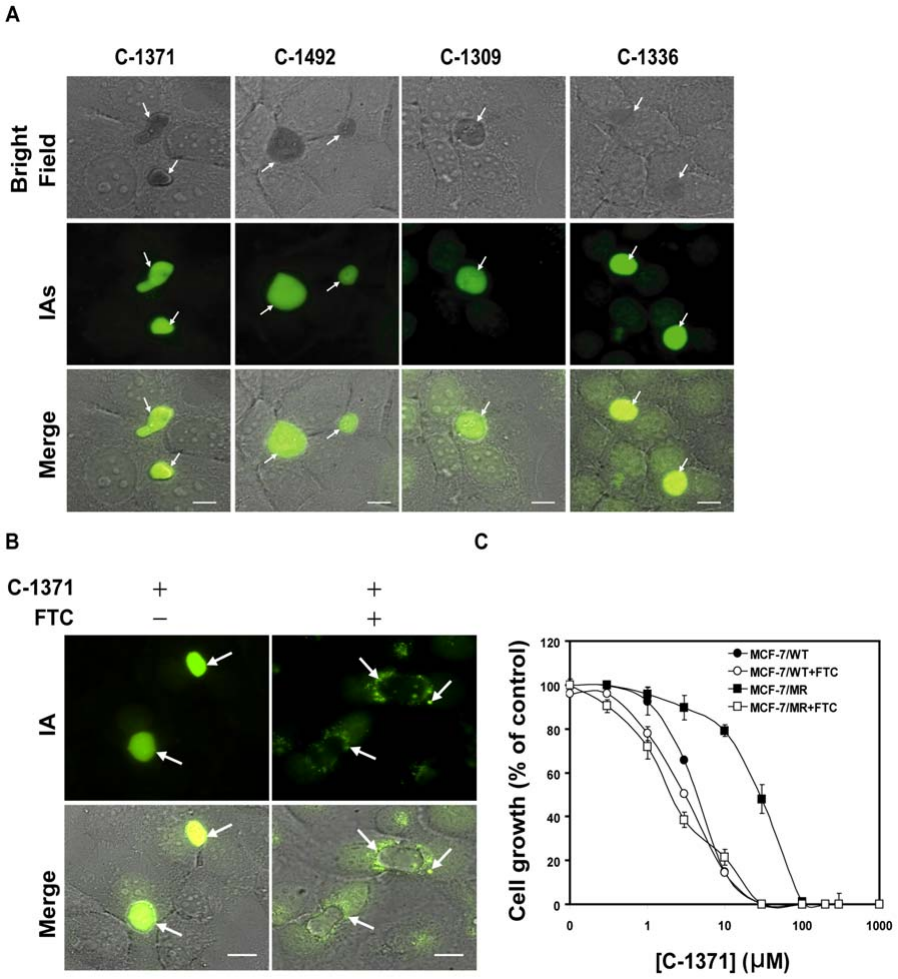
Inhibition of cell growth by IAs and their accumulation in EVs. (A) MCF-7/MR cells were seeded in 6-well dishes (2×10^4^cells/well; 2 ml medium/well) containing glass bottom and grown for 7 days to allow for the formation of EVs. Before adding IAs, cells were provided with riboflavin-deficient medium for at least 48 h to avoid riboflavin autofluorescence. Then, cells were exposed to 5 µM of various IAs for 24 h at 37°C. (B) Cells were pre-treated for one hour with FTC (10 µM) following co-incubation with C-1371 as in panel A. Control cells were cultured in FTC-free medium in the presence of C-1371. Arrows indicate the localization of EVs that lack or contain IAs accumulation. (C) MCF-7 and MCF-7/MR cells were exposed to various concentrations of C-1371 for 72 h in the presence or absence of FTC (10 µM), following which viable cell numbers were determined. Shown are the means of three independent experiments, each performed in triplicates ± SD. The IC_50_ values of C-1371 in MCF-7 and MCF-7/MR cells were 4.3±0.1 and 27.8±3.7, respectively. Throughout the entire study, the bar denotes10 µm.

Recently we have shown that a class of cytotoxic drugs known as IAs including C-1371, C-1492, C-1309 and C-1336 are *bona fide* transport substrates of ABCG2 [12]. We therefore hypothesized that IAs may be actively sequestered and concentrated within ABCG2-rich EVs. Taking advantage of the photosensitizer nature of IAs we examined their intravesicular accumulation in mitoxantrone-resistant breast cancer cells (MCF-7/MR) that overexpress ABCG2 exclusively at the membrane of EVs. MCF-7/MR cells were cultured in riboflavin-deficient medium to avoid riboflavin autofluorescence [9] and incubated with various IAs which are established ABCG2 transport substrates [12]. We found that these IAs were specifically concentrated within ABCG2-rich EVs (**Figure 1A**); sequestration of IAs in EVs was mediated by ABCG2, as it was abolished by the specific ABCG2 transport inhibitor FTC (**Figure 1B**). Moreover, MCF-7/MR cells displayed 7-fold resistance to C-1371, a representative IA; this resistance was fully reversed by FTC (**Figure 1C**).

### Photosensitization of C-1371-accumulating EVs results in rapid destruction of EVs

**Figure 2:**
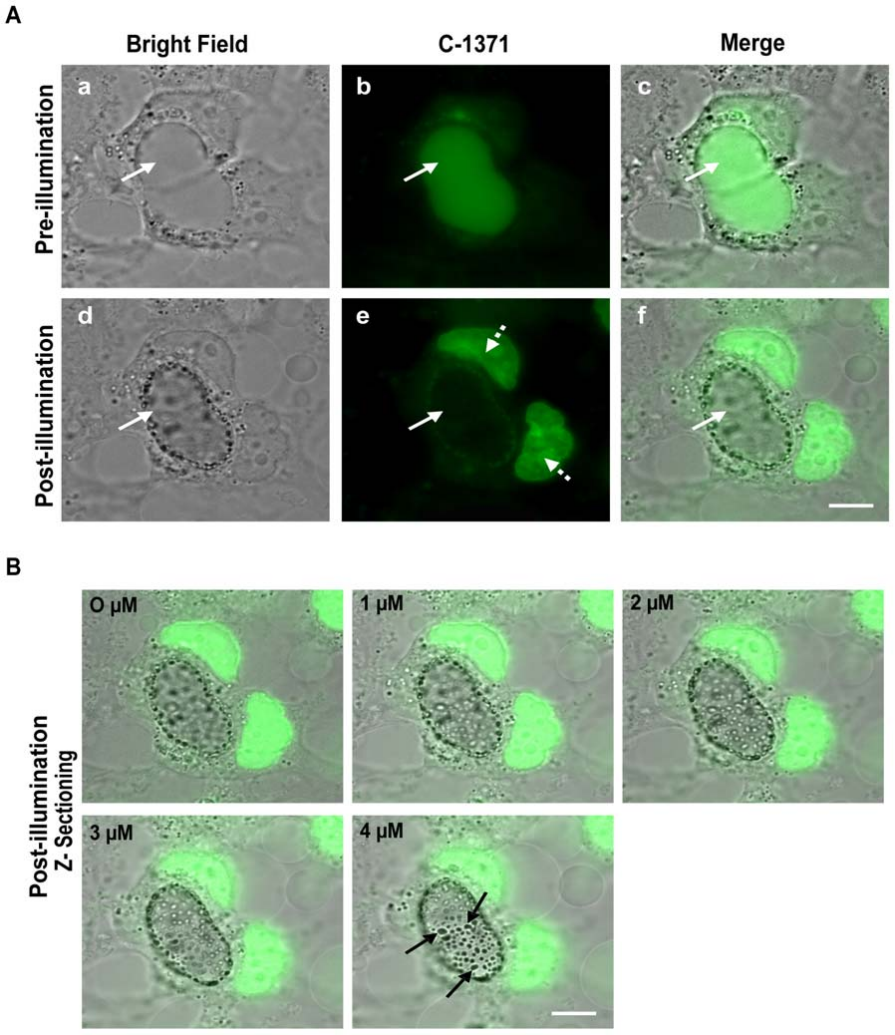
Photosensitization of C-1371-accumulating EVs results in destruction of EVs in MCF-7/MR cells. (A) MCF-7/MR cells were grown as in Figure 1 legend and exposed to C-1371 (10 µM) for 24 h at 37°C to allow for its accumulation within EVs. Then, random fields were photographed using a Zeiss inverted Cell-Observer microscope at an ×630 magnification. Next, these selected fields were continuously exposed to blue light using the GFP+DsRED longpass filter (excitation 470±27; emission 512±20 and 630±98) for 10 min and re-photographed using the same parameters. Continuous arrows point at the location of EVs, whereas dashed arrows point at the nuclei of EVs-forming cells. (B) Following illumination, cells were analyzed by Z-stack sections creating five 1 µm-thick optical slices. The black arrows point at the MSIS formed following photosensitization.

Compartmentalization of IAs in EVs significantly enhanced MCF-7/MR resistance to these drugs [8]. Thus, based on the fluorochrome nature of IAs, we examined whether a PDT approach could overcome the ABCG2-dependent MDR to IAs. To this end, we first determined whether photodestruction of the membrane of EVs occurs in EVs that previously accumulated a photosensitizer IA. MCF-7/MR cells were exposed to C-1371 (10 µM), a representative ABCG2 transport substrate [12], to allow for its accumulation within EVs, following which cells were illuminated (excitation 470±27; emission 512±20 and 630±98). Consistently, EVs highly concentrated C-1371, thereby resulting in increased luminal green fluorescence (**Figure 2A, a–c**). Interestingly, constant exposure of these MDR cells to light for 10 min resulted in a gradual decrease in the fluorescence of EVs and a simultaneous increase in nuclear fluorescence in EVs-forming cells (**Figure 2A, d–f** **and Movie S1**). Moreover, we noted that the oval structure of EVs was severely damaged and deformed, resulting in non-continuous structures with hollow centers; this suggested that exposure to light inflicts severe damage to the membrane of EVs which is shared by EVs-forming cells. Specifically, following illumination, the membrane of EVs ruptured and collapsed, thereby forming multiple small intravesicular structures (MSIS) which were entrapped within the damaged cytoskeletal structure enforcing EVs (arrows in **Figure 2B**). Similar results were obtained with ABCG2-overexpressing flavopiridol-resistant breast cancer cells (MCF-7/FLV1000) which differ from MCF-7/MR cells in their subcellular localization of ABCG2 and frequency of EVs formation ([8] and **Figure S1A**). Exposure of MCF-7/FLV1000 cells to C-1371 resulted in its intravesicular accumulation; illumination of these cells induced the rupture of the EVs membrane, reduction in the volume of EVs and gradual leakage of IAs to the cytoplasm, followed by 1371 intercalation into nuclear DNA, reflected in the appearance of intensely green fluorescent nuclei (**Figure S1 and Movie S2**).

### Immunofluorescence studies of EVs prior to and following photodestruction

**Figure 3:**
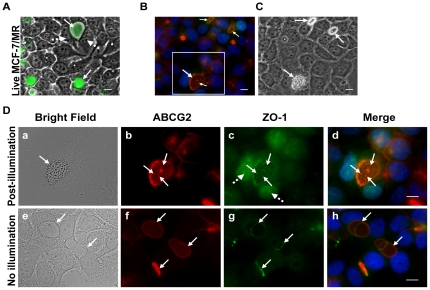
Immunofluorescence studies of ABCG2 and ZO-1 localization following illumination of C-1371-accumulating EVs formed in MCF-7/MR cells. (A) A merged image of bright field and C-1371 green fluorescence in live MCF-7/MR cells before illumination. Immediately after illumination, both the illuminated (B, C and D, a–d) and the non-illuminated (D, e–h) MCF-7/MR cells were fixed in 4% formaldehyde and co-reacted with monoclonal antibodies to ABCG2 (BXP-53) and ZO-1. (B) Illuminated MCF-7/MR cells were stained with ZO-1 (green), ABCG2 (red) and DAPI (blue), at a ×630 magnification. (C)The bright field image underlines the structures of illuminated EVs. (D) An enlarged image of the area is indicated by a white square in panel B. The green nuclear fluorescence in panel C is due to the intercalation of IA into DNA after the rupture of the membrane of EVs due to photosensitization. C-1371-loaded, non-illuminated MCF-7/MR cells served as a control (e–h). Since immunofluorescent staining includes membrane permeabilization, no accumulation of IAs was detected in EVs. Continuous arrows point at EVs whereas dashed arrows point at the nuclei.

**Figure 4:**
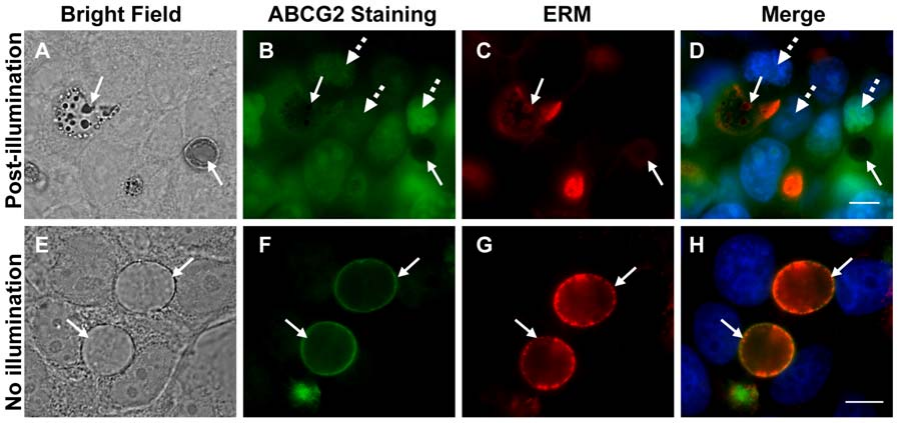
Immunofluorescence studies of ERM and ABCG2 localization following illumination of C-1371-accumulating EVs formed in MCF-7/MR cells. MCF-7/MR cells accumulating C-1371 (10 µM) were either illuminated (excitation 470±27 emission 512±20 and 630±98) for 10 min at a ×400 magnification to allow for photodestruction of EVs (A–D) or not illuminated (E–H). Cells were then immediately fixed and co-reacted with monoclonal antibodies to ABCG2 (BXP-21) and ERM. The green nuclear staining in panel B is due to IA intercalation into nuclear DNA, following EVs photodestruction. Continuous arrows point at EVs whereas dashed arrows point at nuclei.

To characterize the fate of the EVs structure and the intactness of their membrane after illumination, we next studied the structure of C-1371-accumulating EVs after photoexcitation using established EVs membrane markers including ABCG2, ERM and ZO-1. To induce photodestruction of EVs, MCF-7/MR cells harboring C-1371-loaded EVs were first illuminated, fixed and co-stained with antibodies to the established vesicular markers ABCG2 as well as ZO-1, a representative tight junction (TJ)-associated protein that cross-links TJs to the actin-cytoskeleton [21], [22] (**Figure 3**), or with antibodies to ABCG2 and the ERM protein complex (**Figure 4**). ERM proteins are closely related polypeptides linking actin microfilaments to the plasma membrane either directly via binding to the cytoplasmic tail of transmembrane proteins, or indirectly through scaffold proteins attached to membrane proteins [23]. We have previously identified the ERM complex as a structural marker of EVs as it was selectively targeted to the membrane of EVs where it co-localized with ABCG2 (**Figure 4E–H** and [8]). Consistent with our results, we observed a differential compartmentalization of C-1371 prior to and following illumination. Specifically, IAs were highly concentrated in EVs (**Figure 3A**), however upon photoexcitation, IAs leaked out of EVs as the membrane of EVs lysed due to the formation of deleterious ROS including singlet oxygen and hydrogen peroxide [20], resulting in fluorescent nuclei due to DNA-intercalated IAs; clearly, the markedly damaged EVs were free of IAs (**Figure 3D a–d** and **Figure 4A–D**). Moreover, in agreement with our above findings (**Figure 2**), immunofluorescence studies revealed that after illumination, the oval-shaped membrane of EVs ruptured, thereby forming rapidly sealing multiple small vesicular structures termed MSIS which were entrapped within the lumen of the remaining cytoskeletal structure of the highly damaged EVs. Upon rupture of the EVs membrane, proteins associated with the EVs membrane including ABCG2 (**Figure 3D, b**) and ERM (**Figure 4C**) remained adsorbed to the perimeter of the cytoskeletal structure of EVs as well as were present in the membrane of MSIS. Under physiological conditions, TJ proteins surround ABCG2-rich EVs, hence forming intense ring structures precisely at the border of EVs-forming cells, thereby sealing the EVs to the outer environment (**Figure 3d–h**; and [8]). Interestingly, following photodestruction of EVs, ZO-1 was still visible at cell-cell attachment zones surrounding the EVs, similarly to non-illuminated cells (**Figure 3D e–h**); albeit, an intense immunofluorescent signal was also observed in the membrane of MSIS that formed after the rupture of EVs following illumination (**Figure 3D a–d**). IA-accumulating, non-illuminated cells, served as controls to highlight the initial morphology of EVs as well as protein localization (**Figure 3D e–h** and **Figure 4E–H**).

### Dynamics of sub-cellular accumulation of non-ABCG2 substrate IAs and lysosomal photodestruction

**Figure 5:**
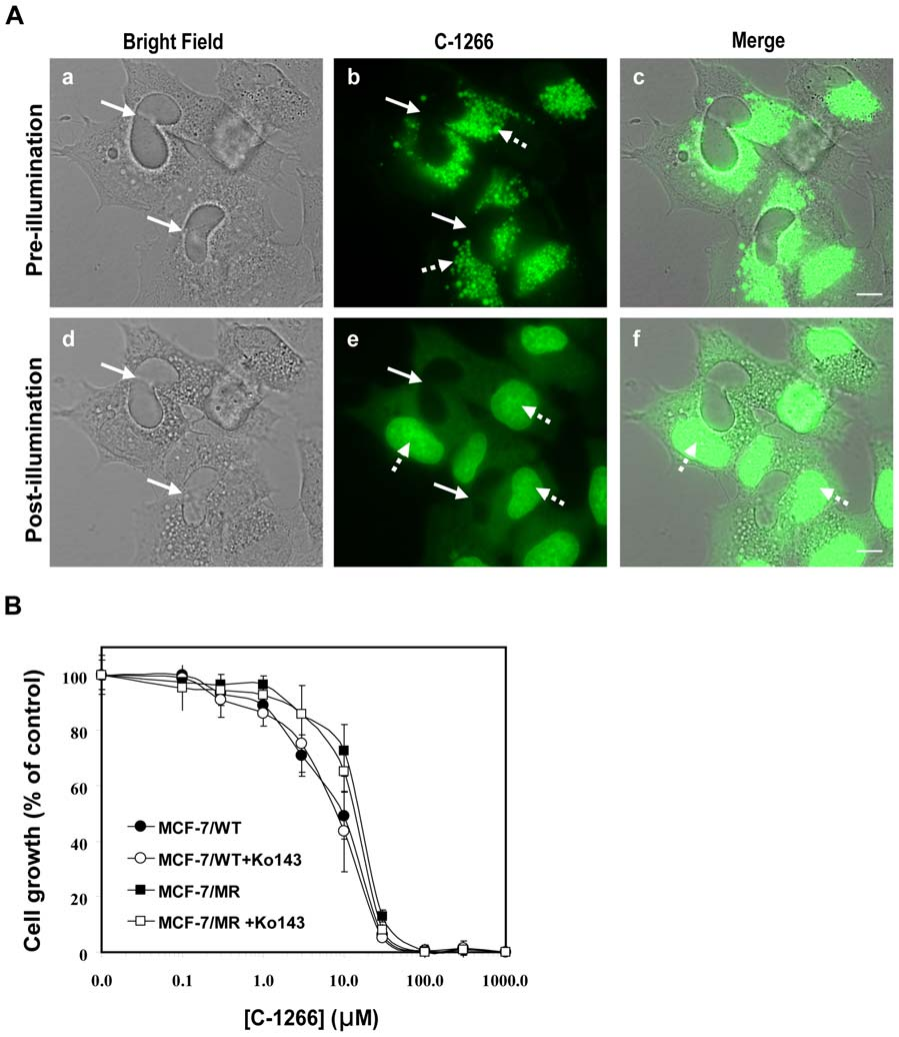
Sub-cellular localization of IAs that are non-ABCG2 substrates and the impact of illumination on cell viability in C-1266-accumulating cells. (A) MCF-7/MR cells were incubated with C-1266 (10 µM), which is not an ABCG2 transport substrate, for 5 h at 37°C (a–c). Selected fields were continuously exposed to blue light for 1–2 min using the Cell-Observer microscope at a ×630 magnification (d–f). Continuous arrows denote the location of EVs that are devoid of IAs (i.e. empty EVs), whereas dashed arrows point at the lysosomal or the nuclear localization of C-1266. (B) MCF-7 and MCF-7/MR cells were exposed to various concentrations of C-1266 for 72 h in the presence or absence of Ko143 (0.7 µM), following which viable cell numbers were determined. Shown are the means of three independent experiments, each performed in triplicates ± SD.

Incubation of MCF-7/MR cells with IAs which are not ABCG2 transport substrates including C-1266 [12], resulted in EVs that were devoid of IA fluorescence, but displayed intense lysosomal fluorescence (**Figure 5A**). Accordingly, pulse exposure of C-1266-rich lysosomes to microscope light resulted in an instantaneous lysosomal rupture with disappearance of fluorescent lysosomes, release of C-1266 to the cytoplasm followed by a gradual increase in nuclear fluorescence (**Figure 5A** and **Movie S3**). Similar results were obtained when MCF-7/MR cells were exposed to the non-ABCG2 substrate C-1375 (data not shown). Previously we have shown that parental MCF-7 cells express little ABCG2 and form much fewer EVs than does their MDR subline, MCF-7/MR [8], [10]. Incubation of MCF-7 cells with IAs which are either ABCG2 substrates (**Figure S2A, upper panel**) or not (**Figure S2B**) resulted in their dramatic accumulation in acidic lysosomes. Photosensitization of these IAs-accumulating lysosomes resulted in their lysis, consequent leakage of the drug to the cytoplasm and gradual accumulation in the nuclei (**Figure S2**). In agreement with this fluorescence microscopy analysis, we observed similar cell sensitivity to C-1266 in MCF-7 and MCF-7/MR cells. Consistent with the suggested lysosomal accumulation of IAs, inhibition of ABCG2 transport function did not alter cellular sensitivity to C-1266 in both of these cell lines (**Figure 5B**). Since MDR cells are known to contain increased numbers of lysosomes per cell, we studied lysosome numbers in parental and MDR cell lines; we determined the number of lysosomes per cell in both MCF-7 and MCF-7/MR cells using the well established viable lysosomal probe Lysotracker red. This live staining revealed a ∼2.5-fold increase in lysosome number in MDR MCF-7/MR cells (3,994±754 fluorescence units/cell) relative to parental MCF-7 cells (1,650±584 fluorescence units/cell). Thus, the increased number of lysosomes in MDR MCF-7/MR cells would contribute to the preferentially increased accumulation of IAs in lysosomes and EVs, hence enhancing the selective photodestruction of MDR cells when compared to normal cells.

### Photoexcitation of IAs that accumulated in EVs markedly increases the cytotoxic effect exerted by IAs

**Figure 6:**
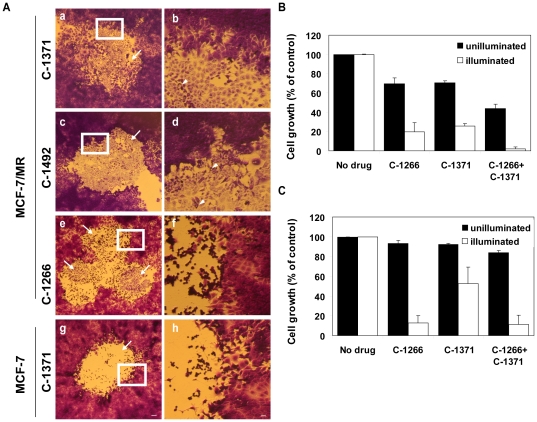
The effect of illumination on the viability and morphology of IAs-accumulating MCF-7 and MCF-7/MR monolayers. (A) Confluent MCF-7/MR (a–f) and MCF-7 (g–h) monolayers were loaded with the indicated IAs (20 µM): 24 h incubation in the case of ABCG2-substrates (i.e. C-1492 and C-1371), and 3 h incubation in the case of non-ABCG2 substrate C-1266. Then, cells were exposed to constant light: 10 min for ABCG2-substrates (a–d) and 2 min for non-ABCG2 substrates (e–f) or while using MCF-7 cells (g–h). The illuminated foci were lysed thus appeared as round, blank areas with cells debris and residual damaged cytoskeletal structures (see arrows). Cells that were not exposed to light surround the illuminated areas and retained their intact morphology. Following illumination, cells were shifted to a fresh medium and incubated for an additional 48 h at 37°C. At the end of the incubation period, cells were fixed with methanol, stained with Crystal Violet, to improve visibility, and analyzed using a Leica binocular under a ×6.4 magnification (a,c,e and g). Bar denotes 100 µm. Enlarged images of the areas indicated by a bold white square, on the left column, were photographed at a ×25.6 magnification and depicted on the right (b,d,f and h). Bar denotes 25 µm. Arrowheads point at the EVs surrounded by cell debris. Confluent MCF-7 (B) and MCF-7/MR (C) monolayers were exposed to the indicated IAs (3 µM) for 72 h, following which cells were exposed to 10 min illumination, whereas control cells were not. Then, growth inhibition was determined as detailed in Materials & Methods.

The results of the current study implied that upon illumination, massive damage is inflicted to the EVs, the membrane of which is shared by EVs-forming neighbor tumor cells. We therefore explored the impact of such photosensitization on cell viability. To this end, EVs-forming MCF-7/MR monolayers were first exposed to a series of IAs that are ABCG2-substrates followed by illumination aimed at inducing photodestruction of EVs. Following an additional incubation for 48 h, cells were fixed with methanol and stained with Crystal Violet to evaluate cell morphology. Light microscopy revealed that the illuminated foci of the monolayers were completely lysed and eradicated, thereby forming blank areas with cell debris and residual cytoskeletal structures surrounded by confluent unaffected monolayer cells that were not illuminated (**Figure 6A**). Thus, cells that were not exposed to light retained their intact morphology. Crystal Violet staining of illuminated MCF-7/MR cells exposed to C-1266 (**Figure 6A e and f**) or parental MCF-7 cells exposed to C-1371 revealed similar results (**Figure 6A g and h**). However, in this case, cell lysis occurred due to lysosomal photodestruction, thereby leaving a symmetric round blank area with completely lysed cells, thus reflecting the exact area exposed to light.

Based on these encouraging results we further hypothesized that ABCG2- overexpressing MCF-7/MR cells may become highly sensitive to the cytotoxic effect exerted by IAs upon illumination, thereby resulting in reversal of MDR. To test this hypothesis, we performed cytotoxicity assays in the absence or presence of illumination. Initially, MCF-7 and MCF-7/MR cells were treated either with C-1266 or C-1371 at a 3 µM concentration which allows high rates of cell survival (C-1266 treatment: 70±6.2% and 93.5±3% in parental and MCF-7/MR cells, respectively; C-1371 treatment: 71±1.8% and 92.4±0.8% in parental and MCF-7/MR cells, respectively) (**Figure 6B and C**). Remarkably, upon illumination, the viability of MCF-7 and MCF-7/MR cells after C-1266 treatment dropped to 19.6% and 12.8%, respectively. Consistently, C-1371 displayed markedly decreased survival rates of 25.6% and 53%, respectively.

### Subcellular localization of topotecan in MDR cancer cells versus drug-sensitive tumor cells and normal breast epithelial cells

**Figure 7:**
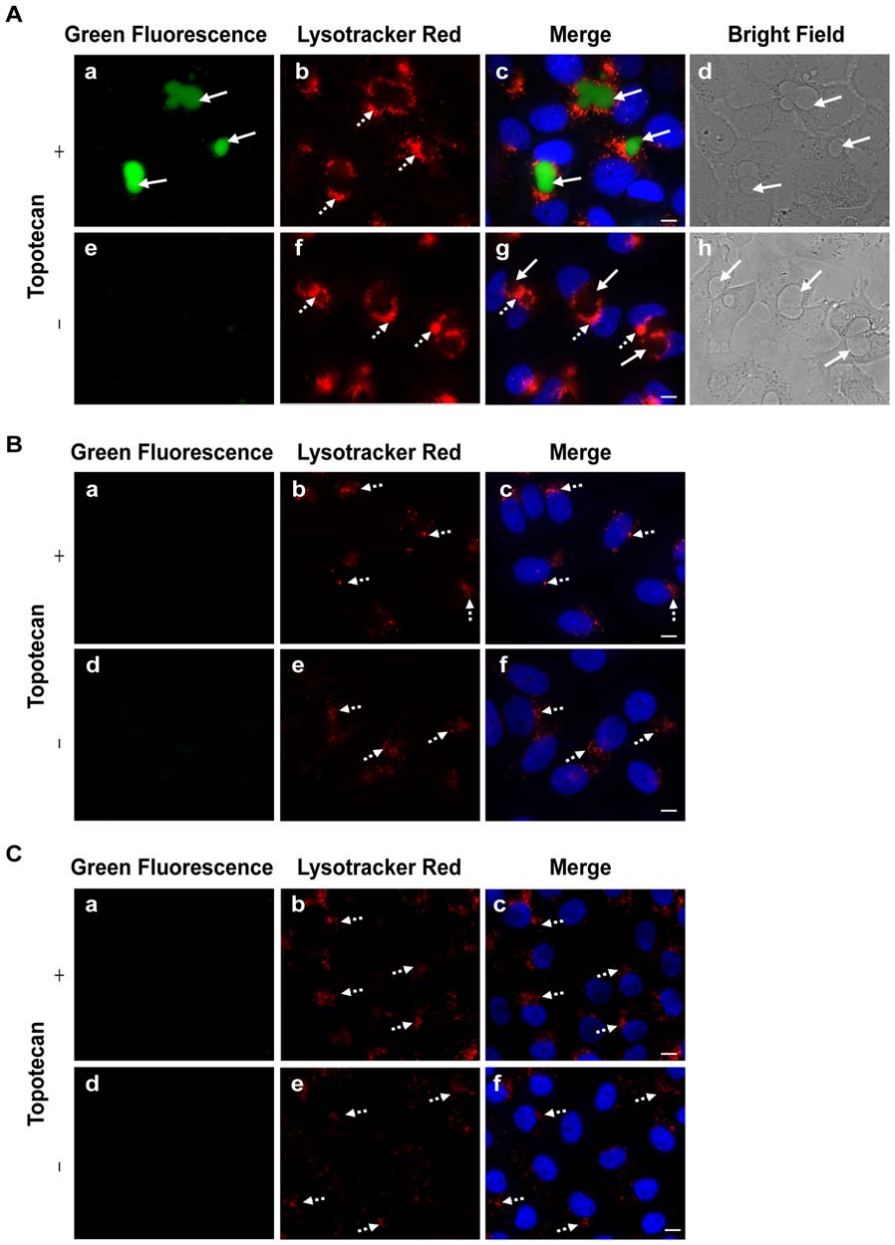
Topotecan accumulates exclusively in EVs formed in MDR cancer cells but not in the lysosomes of MCF-7 or MCF-10A cells. MCF-7/MR (A), MCF-7 (B) and MCF-10A (C) cells were grown in 6-well dishes containing glass bottom in riboflavin-free medium for 5 days to allow for EVs formation in MCF-7/MR cells. Then, cells were incubated with topotecan (1 µM for cancer cells or 0.5 µM for MCF-10A cells) for 24 h at 37°C. Lysotracker red DND99 (100 nM) was added for 1 h prior for cell imaging. Hoechst 33342 (2 µg/ml) was added immediately prior to microscopy analysis and served to visualize nuclei. Cells were then photographed using the Cell-Observer microscope at a ×630 magnification. Bright field images are presented for MCF-7/MR cells to better visualize the location of EVs (d and h A). Cells that were not exposed to topotecan served as a negative control and underline the lysosomal pattern differences (e–h in A; d–f in B and C). Continuous arrows point at topotecan accumulated in EVs (green), whereas dashed arrows point at the lysosomes (red).

**Figure 8:**
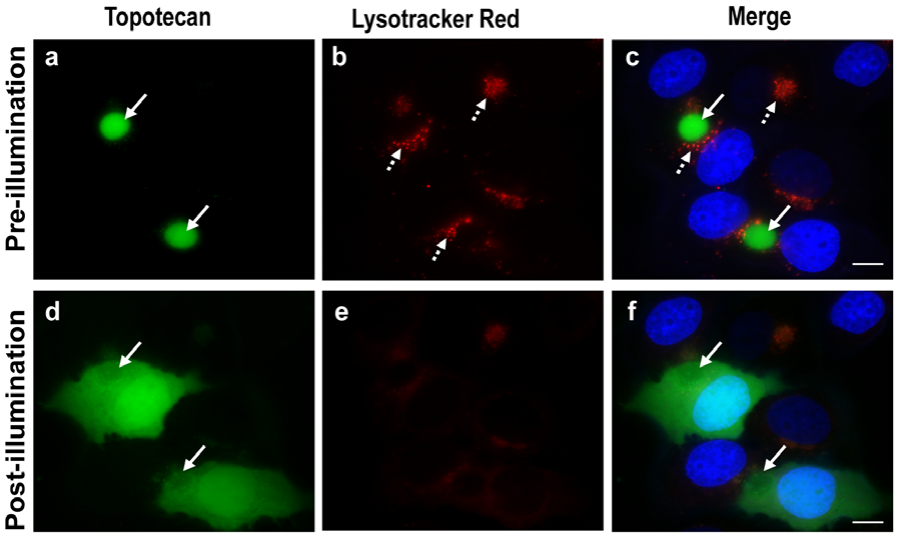
Photosensitization of topotecan-accumulating EVs results in destruction of EVs. MCF-7/MR cells were grown and treated as in Figure 7 and random fields were photographed using the Cell-Observer microscope at a ×630 magnification (a–c). Next, these selected fields were continuously exposed to blue light for 16 min and re-photographed using the same parameters (d–f). Lysosomes were detected using Lysotracker red whereas nuclei were visualized using Hoechst 33342 staining. Continuous arrows point at the location of EVs and dashed arrows at lysosomes.

Our results indicated that IAs may readily accumulate in both EVs via ABCG2-dependent concentration as well as in lysosomes due to the hydrophobic weak base nature of IAs. To further examine the selectivity of the drug treatment with IAs we searched for photosensitive cytotoxic drugs that would localize exclusively in ABCG2-rich EVs but not in lysosomes. In this respect, topotecan, an established ABCG2 transport substrate [8], [15], [16], [17], was highly sequestered in EVs of MDR MCF-7/MR cells with no detectable accumulation in lysosomes (**Figure 7A**). Consistently, no fluorescence signal of topotecan was detected in lysosomes or in nuclei of parental MCF-7 cancer cells (**Figure 7B**). To address the important issue of sub-cellular localization of topotecan in normal breast epithelial cells, we used the non-transformed human mammary epithelial cell line MCF-10A, which is widely used as a normal counterpart of breast cancer cells [24], [25], [26]. MCF-10A cells neither form tumors in nude mice nor colonies in soft agar-based clonogenic assays. Similar to MCF-7 breast cancer cells, no lysosomal accumulation of topotecan was observed in MCF-10A cells (**Figure 7C**). Moreover, illumination of EVs which accumulated topotecan resulted in the rupture of the EVs membrane; this was followed by a gradual leakage of topotecan from EVs to the cytoplasm of EVs-forming cells and its gradual accumulation in nuclei (**Figure 8** and **Movie S4**). The photosensitizing capacity of topotecan was similar to the one obtained with IAs (**Figure 2** and **Figure S1**), albeit topotecan required somewhat longer illumination times to exert its cytotoxic effect, suggesting that topotecan was a less potent photosensitizer than were the studied IAs.

### Alkalinization of lysosomes using bafilomycin A1 abolishes lysosomal drug accumulation, but does not affect drug accumulation in EVs

**Figure 9:**
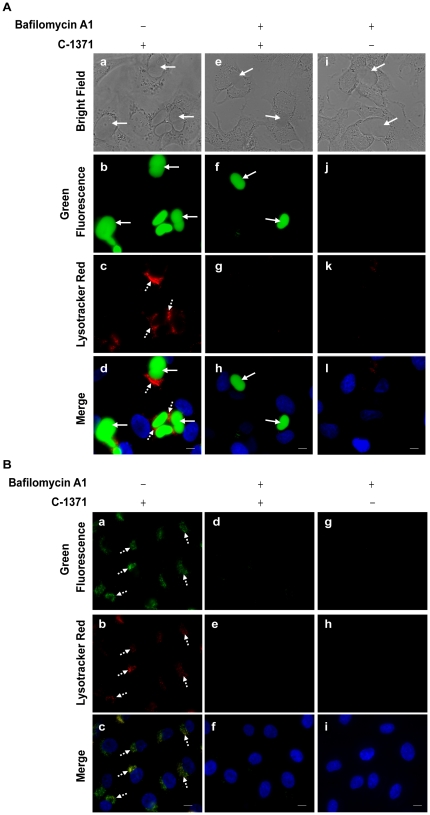
Treatment with bafilomycin A1 abolishes lysosomal accumulation of C-1371, but has no impact on its accumulation in EVs. Cells were grown as described in Figure 7 legend. MCF-7/MR (A) and MCF-10A (B) cells were exposed to C-1371(10 µM) for 24 h or 5 h, respectively, in the absence or presence of bafilomycin A. Lysosomes and nuclei were visualized as described in Figure 7 legend. Cells were then photographed using the Cell-Observer microscope at an ×630 magnification. Cells exposed solely to bafilomycin A1 served as negative controls (i–l in A; g–I in B). Continuous arrows point at the EVs accumulation of C-1371 (green), whereas the dashed arrows point at the lysosomes (red).

Applying a PDT approach using IAs which markedly concentrate within lysosomes that undergo effective lysosomal photodestruction might be toxic to normal tissues that surround the malignant tumor. To further explore this drug treatment selectivity, we postulated that the acidic pH of lysosomes is the driving force for the marked lysosomal compartmentalization of IAs. Hence, we used bafilomycin A1, a potent inhibitor of H^+^-ATPase (i.e. vesicular ATPase) to alkalinize lysosomal pH. MCF-7/MR cells were incubated with C-1371 (10 µM), an established ABCG2 transport substrate, for 24 h with or without pre-incubation with bafilomycin A1 (100 nM). Consistent with our previous findings, C-1371 highly accumulated in EVs due its ABCG2-dependent concentration (**Figure 9A a–d**). Following pre-incubation with bafilomycin A1, no lysosomal accumulation of C-1371 was detected, whereas accumulation of C-1371 in EVs was completely retained (**Figure 9A e–h**). Normal breast epithelial MCF-10A cells were exposed to C-1371 (10 µM) for 5 h; expectedly, C-1371 readily accumulated in lysosomes and co-localized with Lysotracker red (**Figure 9B a–c**). Pre-incubation of MCF-10A cells with bafilomycin A1 resulted in the complete elimination of the lysosomal signal as visualized by Lysotracker red along with the complete disappearance of lysosomal localization of C-1371 (**Figure 9B d–f**). MCF-7/MR (**Figure 9A i–l**) and MCF-10A cells (**Figure 9B g–i**) incubated solely with bafilomycin A1 served as positive controls to underline the effect of bafilomycin A1. Hence, these results suggest that lysosomal accumulation of IAs can be abolished by lysosomal alkalinizing agents without compromising the marked accumulation of the photosensitizer in EVs. The latter could be effectively harnessed to selectively kill MDR cancer cells via photodestruction of EVs without harming normal cells due to the lack of lysosomal drug accumulation.

## Discussion

In the current paper we describe a novel pharmacological lethal Trojan horse strategy to differentially kill MDR breast cancer cells via photoexcitation of photosensitizer anticancer drugs that highly accumulated in ABCG2-rich EVs. Towards this end, we provide several lines of evidence supporting the conclusion that IAs which enter cells by diffusion, undergo a dramatic compartmentalization within ABCG2-rich EVs in an ABCG2-dependent manner, similarly to topotecan [8]. First, hydroxyl group-containing IAs which proved to be *bona fide* ABCG2 transport substrates [12], highly accumulated within EVs. Consistently, the IAs C-1266 and C-1375, which lack a hydroxyl group and thus are not ABCG2 transport substrates, did not accumulate in EVs. Instead, as hydrophobic weak bases, these IAs highly accumulated in acidic lysosomes presumably due to irreversible protonation. Consistently, inhibition of ABCG2 transport function by the specific transport inhibitor Ko143 did not alter the cytotoxicity (IC_50_ values) of the non-ABCG2 substrates C-1266 and C-1375 in both parental MCF-7 and MDR MCF-7/MR cells. Second, the accumulation of hydroxyl group-containing IAs within EVs was completely abolished by ABCG2 transport inhibitors. Third, the compartmentalization within EVs of IAs which are ABCG2 transport substrates, away from their intracellular targets, resulted in MDR. Indeed, MCF-7/MR cells displayed 7-fold resistance to C-1371, an ABCG2 transport substrate.

Taking advantage of the high level sequestration of these photosensitizer IAs within EVs, we show here that upon illumination, EVs underwent rapid lysis, thereby resulting in selective killing of these MDR cells. Specifically, following photosensitization, the membrane of EVs ruptured thus forming multiple fragments of the EVs membrane; these fragments immediately sealed and appeared as MSIS which appeared entrapped within the lumen of the remaining cytoskeleton of the highly damaged EVs. Consequently, no IAs-based green fluorescence was retained in the lumen of these MSIS. These results suggest that upon photosensitization, IAs-loaded EVs release their IAs content due to the lysis of the EVs membrane with consequent formation of MSIS. Moreover, it is highly likely that the lumen of the EVs and MSIS is devoid of ATP, hence excluding the possibility of residual functional ABCG2 activity.

We have previously shown that the formation of various ROS upon illumination of IAs, is the underlying basis for IAs-dependent cellular photodestruction [20]. We therefore propose here that the cellular damage inflicted by illumination resulting in lysis of the membranes of both IAs-loaded EVs and lysosomes and consequent cell death is mediated by rapid production of ROS. This conclusion is supported by the following line of evidence: first, in a recent study we used established methodologies to show that photoexcitation of various IAs results in the formation of ROS including singlet oxygen and hydrogen peroxide [20]. Second, the mediation of cell kill by ROS in our IA-loaded MDR cells undergoing lysosomal photodestruction, is in accord with the widely accepted mechanism of cytotoxicity of various photosensitizers used in PDT including protoporphyrins; upon illumination, these photosensitizers undergo fluorescence excitation (i.e. the triplet excited state), hence immediately transferring their fluorescence (i.e. energy) to molecular oxygen, thereby resulting in an instantaneous production of singlet oxygen and ROS. Recent studies have determined that ROS production in a PDT scheme using photoexcited protoporphyrin IX induced an essentially instantaneous production of ROS with a remarkable time rate constant of 0.0044 min^−1^ [27]. Thus, the rapid lysosomal photodestruction in our MDR tumor cells here is consistent with this rate constant of ROS generation. Singlet oxygen radicals and ROS are extremely harmful to biomolecules as they oxidize amino acids in proteins, polyunsaturated fatty acids in lipids (i.e. lipid peroxidation), factors essential for enzymes, thereby resulting in rapid cell death [28]. As such, upon photoexcitation, the abrupt lysis of IAs-loaded EVs and lysosomes is consistent with the rapid generation of ROS. Moreover, illumination of EVs that are not containing photosensitizers has no deleterious effect on cell morphology and viability, thus the cytotoxic effect achieved is due to an intrinsic photosensitizing activity of IAs that highly accumulate in the lumen of ABCG2-rich EVs.

In the absence of ATP-driven ABC transporters which efficiently concentrate hydroxyl group-bearing IAs in EVs and in the case of non-ABCG2 substrates, the organelles that predominantly compartmentalized IAs were acidic lysosomes. Based on the co-staining of IAs with the well established viable lysosomal probe Lysotracker red, we found here that the subcellular accumulation of IAs is similar to the well documented [29], [30] localization of hydrophobic weak base chemotherapeutics such as anthracyclines, *Vinca* alkaloids and other cytotoxic agents including acridone-based fluorescent compounds, the latter of which are structurally related to IAs. Nevertheless, at higher concentrations, IAs can also reach the nucleus where they readily bind DNA and potently inhibit topoisomerase II activity [11]. Upon photoexcitation of IAs-loaded lysosomes, ROS formation occurs which results in an instantaneous rupture of lysosomes and consequent release of the highly acidic content of lysosomes including a wide spectrum of destructive hydrolytic enzymes. Such lysosomal lysis leads to rapid digestion of polypeptides and cytoskeletal structures, nucleic acids and biomembranes and consequent cell lysis. This conclusion is supported by the Crystal Violet staining of monolayer cells undergoing photodestruction, which displayed clear border between cell debris with residual cytoskeletal scaffolds of lysed cells that were illuminated and intact cells which were non-illuminated. Moreover, cytotoxicity studies revealed that MDR MCF-7/MR cells became highly sensitive to the cytotoxic effect exerted by IAs upon illumination, hence resulting in reversal of MDR.

PDT acts directly on tumor cells typically via occlusion of the vasculature that nourishes the tumor [31]. Treatment selectivity is based on the accumulation of higher photosensitizer levels within the target tumor than in the surrounding normal tissues. ABCG2 overexpression in the plasma membrane of malignant tumors that mediates the extrusion of multiple anticancer drugs from the tumor cells can decrease both the efficacy and selectivity of MDR type drugs [31], [32]. In contradistinction, ABCG2 overexpression and its specific targeting to the EVs membrane of breast, lung and hepatic epithelial malignant tissues allows for the high level concentration of photosensitizer within EVs, thereby enhancing the selectivity and efficacy of these PDT agents, as was shown here by photosensitization of IAs- and topotecan-loaded EVs. Hence, we propose five distinct mechanisms that support the specific targeting of MDR cells via photodestruction of EVs and lysosomes as a pharmacologically lethal Trojan horse approach: First, ABCG2-rich EVs are present solely in MDR cells but not in non-MDR cancer cells (e.g. MCF-7 cells) or in normal cells from healthy tissues (e.g. MCF-10A). Second, the markedly increased number of lysosomes in ABCG2-overexpressing MDR MCF-7/MR cells and in various other MDR cancer cells relatively to their drug naïve cancer cells as well as normal tissues, renders these MDR cells much more vulnerable to lower light intensities due to a substantial increase in lysosomal sequestration of photosensitizers. These decreased light intensities should be much less deleterious to normal tissues that accumulate markedly less photosensitizers. Third, the frequently increased acidity of lysosomal pH in MDR cells along with the parallel alkalinization of their cytoplasm [33], [34], [35] should further enhance the differential photodestruction of MDR cells but not healthy tissues. Forth, and perhaps most importantly, in order to maximize the selectivity of the proposed PDT treatment one can rationally select a photosensitizer that is an ABCG2 transport substrate but is not a hydrophobic weak base, thus ruling out lysosomal drug sequestration and subsequent lysosomal photodestruction upon illumination of normal tissues surrounding the malignant tumor. In this respect, we showed here that topotecan fulfilled the above requirements; topotecan accumulated exclusively in EVs of MDR cells but not in lysosomes of non-MDR cancer cells (MCF-7) or in normal breast epithelial cells (MCF-10A) and effectively mediated photodestruction of EVs. Fifth, an additional future approach to improve treatment specificity is to combine hydrophobic weak base photosensitizers and well tolerated lysosomal alkalinizing agents such as bafilomycin A1. This drug combination treatment allows for drug compartmentalization within EVs with no residual lysosomal accumulation thus eliminating the lysosomal photodestruction route, thereby allowing for selective targeting of MDR cancer cells.

In summary, targeting MDR cells via photodestruction of EVs and lysosomes can be used as a selective pharmacologic lethal Trojan horse approach to overcome MDR. A photosensitizer can be rationally designed and/or chosen to accumulate solely in EVs (e.g. topotecan), in lysosomes (IAs that are non-ABCG2 transport substrates) or in both EVs and lysosomes (e.g. IAs that are ABCG2 transport substrates). Further studies are warranted to optimize the selectivity and applicability of such a pharmacologic Trojan horse approach to selectively target and eliminate MDR cancers while minimizing toxicity to normal tissues.

## Materials and Methods

### Chemicals

Fumitremorgin C (FTC), mitoxantrone, topotecan, Hoechst 33342 and DAPI were purchased from Sigma-Aldrich (St. Louis, MO). Lysotracker red DND99 was from Invitrogen (Carlsbad, CA). Bafilomycin A1 was purchased from Enzo Life science. Imidazoacridinones were synthesized by Prof. M. Cholody, B. Horowska and M. Konieczny and kindly provided by Prof. A. Skladanowski, Gdansk University, Gdansk, Poland.

### Tissue culture

Human breast cancer MCF-7 cells [7], their mitoxantrone-resistant subline MCF-7/MR as well as flavopiridol-resistant MCF-7/FLV1000 cells were grown as previously described [8], [10]. The immortalized, non-transformed human mammary epithelial cell line MCF-10A, was grown in complete growth medium composed of DMEM/F12 (Biological Industries, Beth-Haemek, Israel) supplemented with 5% fetal calf serum (Invitrogen, Carlsbad, CA), 10 µg/ml insulin (Biological Industries, Beth-Haemek, Israel), 0.5 µg/ml hydrocortisone, 20 ng/ml epidermal growth factor (EGF) and 100 ng/ml cholera toxin (Sigma, St. Louis, MO), glutamine and antibiotics (Biological Industries, Beth-Haemek, Israel). Prior to vesicular drug accumulation experiments, cells were grown in custom-made riboflavin-deficient RPMI-1640 medium (Biological Industries, Beth-Haemek, Israel) supplemented with 10% dialyzed fetal calf serum (Invitrogen, Carlsbad, CA), glutamine and antibiotics. All cell lines were grown in a 5% CO_2_-humidified incubator at 37°C.

### Live cell imaging

Cells were seeded in 24-wells plates on sterile glass coverslips (5×10^3^cells/2 ml) or in dishes containing cover glass bottom (2×10^4^cells/2 ml; World Precision Instruments) and grown in riboflavin-free RPMI-1640 medium for at least 72 h prior to the addition of drugs. Cells were then incubated with various IAs or with topotecan for the indicated times at 37°C. In order to alkalinize lysosomes, cells were pre-incubated with the H^+^-ATPase inhibitor bafilomycin A1 (100 nM) for 1 h followed by an additional co-incubation with the indicated photosensitizer. Lysotracker red DND99 (100 nM) was added 1 h prior to microscope imaging to follow lysosomes in viable cells. Hoechst 33342 (2 µg/ml) served as a viable DNA dye to follow nuclei. In all live imaging microscopy experiments, before analysis, cells were washed thrice with PBS, resuspended in PBS supplemented with 1 mM CaCl_2_, 1 mM MgCl_2_ and 10 mM D-glucose. Then, random colonies were analyzed using Zeiss inverted Cell-Observer microscope, equipped with a CO_2_ and 37°C chamber, using the following filters: phase mode, HE GFP (excitation and emission at 470±40 and 525±50 nm, respectively), GFP+DsRED longpass filter (excitation 470±27 and emission 512±20 and 630±98) or DAPI mode (excitation and emission at 365 and 445±50 nm, respectively) at a magnification of ×400–×630. The merged images were generated using the Cell-Observer software.

### Photosensitization procedure

For drug accumulation experiments, different incubation times were used i.e. 24 hours for intra-EVs accumulation versus 3–5 hours for lysosomal accumulation. These incubation times were selected as the experimentally proven minimal times necessary to obtain optimal fluorescence intensity, which in turn is sufficient to exert a cytotoxic effect following illumination. Drug-loaded cells were washed with PBS, resuspended in PBS supplemented with 1 mM CaCl_2_, 1 mM MgCl_2_ and 10 mM D-glucose and immediately photographed prior to photoexcitation. Next, cells were photosensitized using the Zeiss inverted Cell-Observer microscope equipped with the GFP+DsRED longpass filter (excitation 470±27 and emission 512±20 and 630±98) at ×400 or ×630 magnification. The exposure to the constant blue light was then imaged. In some cases the multiple snap-shots were animated into a video using LSM image browser software (Zeiss Inc.). Photodestruction of IAs-loaded EVs required a continuous illumination for approximately 6–10 min. The different times needed for photodestruction of different EVs were due to the variable volume of EVs, drug concentration in the extracellular medium and ABCG2 expression levels at the membrane of individual EVs. Photodestruction of topotecan-loaded EVs required longer illumination period of approximately 16 min. Lysosomal photodestruction occurred rapidly, within a time frame of 2–3 min. Longer exposure times to light had no additional deleterious effect on cellular morphology or viability.

### Immunofluorescence analysis

Cells were seeded on sterile glass coverslips in 24-well dishes (5×10^4^cells/2 ml) in riboflavin-deficient medium and grown for 7 days at 37°C to allow for the formation of multiple EVs. Immunofluorescence analysis was performed as previously described [8]. Specifically, ABCG2 was visualized using either BXP-21 or BXP-53 monoclonal antibodies (at 1∶100 dilution, a generous gift of Dr. G.L. Scheffer and Prof. R.J. Scheper), followed by incubation with the secondary antibodies: FITC-conjugated donkey anti-mouse IgG or rhodamine red-conjugated donkey anti-rabbit antibody (1∶100 dilution, Jackson ImmunoResearch Laboratories, West Grove, PA), respectively. ZO-1 was visualized using a mouse anti-ZO-1 monoclonal antibody (1∶25 dilution, Invitrogen, Carlsbad, CA). The ERM protein complex was visualized using rabbit monoclonal anti-ERM antibody (1∶500 dilution, Epitomics, Burlingame, CA), which detects all three ERM proteins. Cell nuclei were counterstained with the DNA dye DAPI (0.5 µg/ml) during the incubation with the secondary antibody. Samples were examined using Zeiss inverted Cell-Observer. Merged images were obtained using the AxioVision program (Zeiss, version 4.7).

### Crystal Violet staining

Cells were grown to full confluence, exposed to 20 µM C-1266 for 2 h or to C-1371 and C-1492 for 24 h at 37°C and photoexcited at random spots in the monolayer as described above under “Photosensitization procedure”. Cells were then washed and resuspended with standard medium and incubated for an additional 48 h at 37°C. At the end of incubation period, cells were washed twice with PBS, fixed with 70% methanol for 1 min, re-washed and stained with Crystal Violet (diluted 1∶5, Merck). The Crystal Violet dye served to obtain a contrast for better visualization of cells and cell debris. This staining was executed by applying the dye to the cells monolayer for approximately 1 min following three washes with double distilled water. Then, cells were dried and imaged using Leica M80 binocular (Leica microsystem GmbH, Wetzlar, Germany) at the indicated magnifications.

### Cytotoxicity assays

The cytotoxic activity of antitumor agents was determined using the colorimetric XTT cell proliferation kit (Biological Industries, Beth-Haemek, Israel). Parental MCF-7 and MCF7/MR cells were seeded (2×10^3^–5×10^3^ cells/well) in 96-wells plates and grown for 3 days to allow for the formation of EVs. Cells were then subjected to increasing concentrations of IAs, incubated for 72 h in the absence or presence of the ABCG2 transport inhibitors: FTC (10 µM) or Ko143 (0.7 µM), following which viable cell numbers were determined using the instructions of the manufacturer. To determine the effect of illumination, IAs- treated cells were photosensitized (excitation 470±27 for 10 min) and incubated for an additional 48 h prior to determination of growth inhibition using the XTT assay. All experiments were performed in riboflavin-deficient medium. Drug concentrations required to inhibit cell growth by 50% (IC_50_) were determined and compared between the cell lines. Drug resistance levels (fold) were calculated by dividing the values of IC_50_ of MCF-7/MR cells by that of parental MCF-7 cells or the IC_50_ of non-illuminated cells by that of illuminated cells.

## Supporting Information

Figure S1 **IAs accumulation and photodestruction analysis in flavopiridol-resistant breast cancer MCF-7/FLV1000 cells.** (A) Merged image of fixed MCF-7/FLV1000 cells stained with monoclonal antibody to ABCG2 (BXP-21) and DAPI. (B) Live MCF-7/FLV1000 cells were incubated with C-1371 (10 µM) for 24 h at 37°C and illuminated for 10 min. Continuous arrows denote the location of IAs in EVs, whereas dashed arrows point at the nuclei. Immunofluorescence and live cell analysis was performed using a Zeiss inverted Cell-Observer microscope at a ×630 magnification.
(TIF)

Figure S2 **In absence of EVs, both classes of IAs accumulate in lysosomes which undergo rapid photodestruction upon illumination.** (A) Parental MCF-7 cells were grown in 6-well dishes containing glass bottom and exposed to C-1371 (10 µM) for 2 h at 37°C. Lysotracker red DND99 (100 nM) was added for 1 h prior to fluorescence imaging. Cells were then photographed using the Cell-Observer microscope at an ×630 magnification. Then selected fields were constantly illuminated and photographed every second for a total duration of 1–3 min using the same parameters. Continuous arrows point at the C-1371 accumulating lysosomes, whereas the dashed arrows point at the nuclei. (B) MCF-7 cells were grown, treated and analyzed as in A, but exposed to C-1266. Shown is the localization of C-1266 during the time course of illumination. Presented are selected time points including 0, 30, 50 and 60 sec. Arrows denote the dynamics of time-dependent lysosomal photodestruction.
(TIF)

Movie S1 **Imaging of photodestruction of EVs and gradual nuclear accumulation of C-1371 upon illumination of MCF-7/MR cells.** MCF-7/MR cells were grown as described in Figure 2 legend and exposed to C-1371 (10 µM) for 24 h at 37°C to allow for its accumulation within EVs. Then, random fields were continuously exposed to blue light using the GFP+DsRED longpass filter and photographed each 5 sec for 120 cycles (a total of 10 min) using the ted Cell-Observer microscope at a ×630 magnification. The obtained time-lapse images were animated into a video using LSM image browser software (Zeiss Inc.).
(MOV)

Movie S2 **Photodestruction of EVs and gradual nuclear accumulation of C-1371 upon illumination of MCF-7/FLV1000 cells.** MCF-7/FLV1000 cells were exposed to C-1371 (10 µM) for 24 h at 37°C to allow for its accumulation within EVs. Then, random fields were continuously exposed to blue light using the GFP+DsRED longpass filter and photographed each 5 seconds for a total of 120 cycles (a total of 10 min) using the Cell-Observer microscope at a ×630 magnification. The obtained time-lapse images were animated into a video using LSM image browser software (Zeiss Inc.).
(MOV)

Movie S3 **Lysosomal photodestruction and gradual nuclear accumulation of C-1266 upon illumination.** MCF-7/MR cells were grown as in Figure 5 legend and exposed to C-1266 (10 µM) for 5 h at 37°C. Then, random monolayer fields were illuminated (excitation 470±27 emission 512±20 and 630±98) for 1 min and photographed every sec using the Cell-Observer microscope at a ×630 magnification. The obtained time-lapse images were animated into a video using LSM image browser software (Zeiss Inc.). Shown is an identical field to Figure 5B.
(MOV)

Movie S4 **EVs that accumulate topotecan undergo photodestruction upon illumination of MCF-7/MR cells.** MCF-7/MR cells were grown and treated as described in Figure 8 legend. The continuous exposure to blue light using the GFP+DsRED longpass filter was photographed each 5 sec for 200 cycles (a total of 16 min) using the Zeiss inverted Cell-Observer microscope at a ×630 magnification. The obtained time-lapse images were animated into a video using LSM image browser software (Zeiss Inc.).
(MOV)
