# Supplementary figures and images for: Overcoming Multidrug Resistance via Photodestruction of ABCG2-Rich Extracellular Vesicles Sequestering Photosensitive Chemotherapeutics
Source: PLoS One. 2012 Apr 18;7(4):e35487. doi: 10.1371/journal.pone.0035487 (PMC3329466; doi:10.1371/journal.pone.0035487)

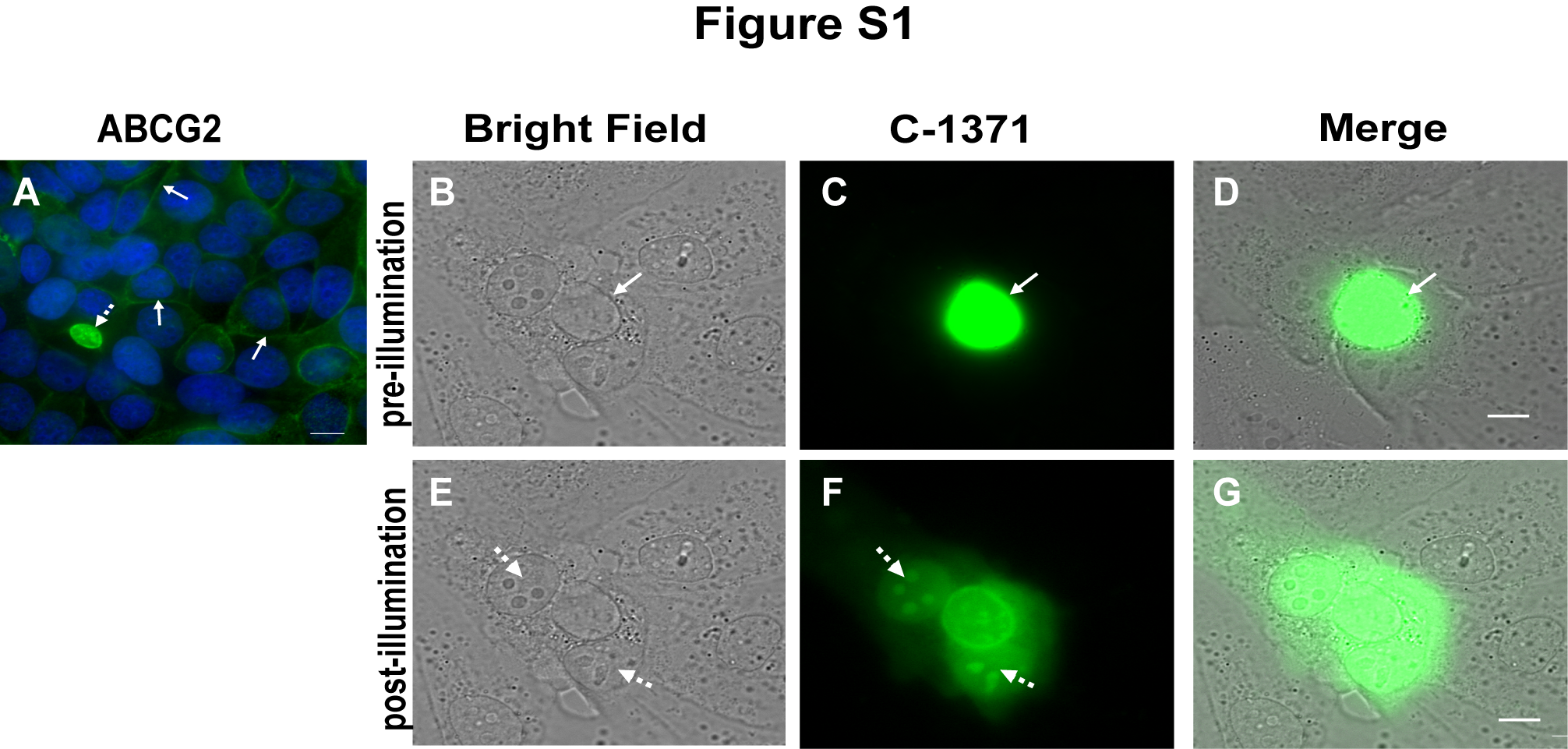

Supplement: Figure S1 — IAs accumulation and photodestruction analysis in flavopiridol-resistant breast cancer MCF-7/FLV1000 cells. (A) Merged image of fixed MCF-7/FLV1000 cells stained with monoclonal antibody to ABCG2 (BXP-21) and DAPI. (B) Live MCF-7/FLV1000 cells were incubated with C-1371 (10 µM) for 24 h at 37°C and illuminated for 10 min. Continuous arrows denote the location of IAs in EVs, whereas dashed arrows point at the nuclei. Immunofluorescence and live cell analysis was performed using a Zeiss inverted Cell-Observer microscope at a ×630 magnification. (TIF) [file pone.0035487.s001.tif]

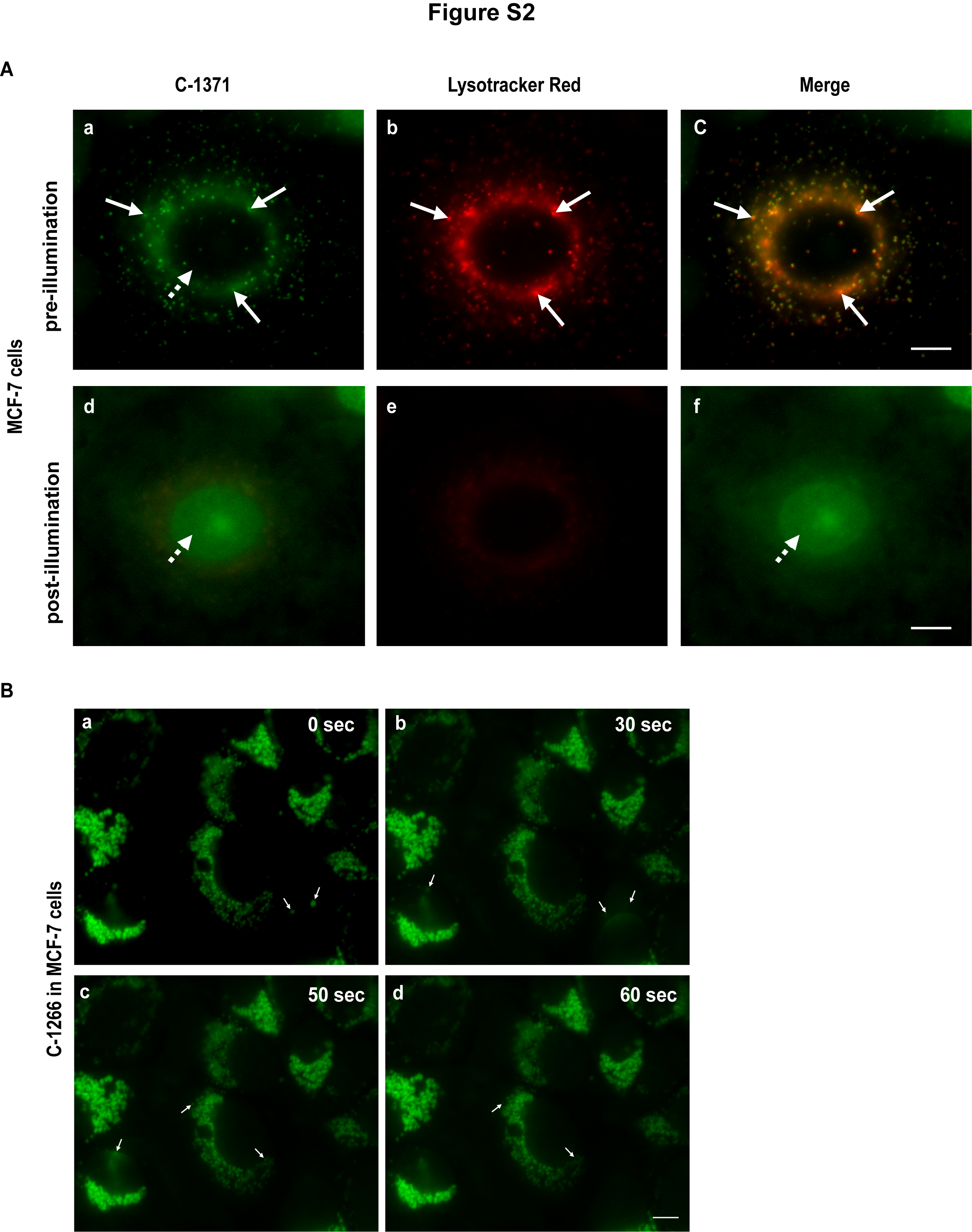

Supplement: Figure S2 — In absence of EVs, both classes of IAs accumulate in lysosomes which undergo rapid photodestruction upon illumination. (A) Parental MCF-7 cells were grown in 6-well dishes containing glass bottom and exposed to C-1371 (10 µM) for 2 h at 37°C. Lysotracker red DND99 (100 nM) was added for 1 h prior to fluorescence imaging. Cells were then photographed using the Cell-Observer microscope at an ×630 magnification. Then selected fields were constantly illuminated and photographed every second for a total duration of 1–3 min using the same parameters. Continuous arrows point at the C-1371 accumulating lysosomes, whereas the dashed arrows point at the nuclei. (B) MCF-7 cells were grown, treated and analyzed as in A, but exposed to C-1266. Shown is the localization of C-1266 during the time course of illumination. Presented are selected time points including 0, 30, 50 and 60 sec. Arrows denote the dynamics of time-dependent lysosomal photodestruction. (TIF) [file pone.0035487.s002.tif]
